# Agonistic 4-1bb antibodies in combination with inhibitory antibodies against CTLA-4, PD-L1 or LAG-3 ACT on CD8+ T cells in the tumor microenvironment and synergize to promote regression of established tumors

**DOI:** 10.1186/2051-1426-2-S3-P213

**Published:** 2014-11-06

**Authors:** Brendan Horton, Stefani Spranger, Jason Williams, Thomas Gajewski

**Affiliations:** 1 Committee on Cancer Biology University of Chicago, Chicago, IL, USA; 2 Department of Pathology University of Chicago, Chicago, IL, USA; 3 Committee on Immunology University of Chicago, Chicago, IL, USA

## 

Tumors can be placed into two categories: those that are T cell-inflamed and those that are not, based on specific chemokine and type I interferon gene expression signatures and CD8^+ ^tumor infiltrating T cells (TIL). That these immune-inflamed tumors are not destroyed by the CD8^+ ^TIL argues that mechanisms must be present to render the CD8^+ ^TIL dysfunctional. Baseline infiltration of CD8^+ ^T cells appears to be critical for tumor regression in response to neutralizing antibodies against CTLA-4 and PD-L1, indicating that these immunotherapies work on T cells in the tumor microenvironment. To better characterize the functional properties and molecular targets in dysfunctional TIL, we have applied knowledge from in vitro T cell anergy studies towards an analysis of T cells in the tumor microenvironment using several mouse tumor models. These experiments led to the finding that dysfunctional CD8^+ ^TIL express the inhibitory receptors PD-1 and LAG-3, but also paradoxically the co-stimulatory molecules 4-1BB and OX-40. We therefore investigated whether delivering positive signals through costimulatory receptors, in combination with blockade of specific inhibitory receptors, could act on dysfunctional TIL to restore their function and induce tumor regression. We found that an agonistic anti 4-1BB antibody combined with inhibitory antibodies against either PD-L1, LAG-3 or CTLA-4 induce tumor regression of established B16.SIY tumors. To test whether these combinations affect CD8^+ ^T cells in the tumor microenvironment or in the periphery we measured the frequency of SIY reactive CD8^+ ^T cells in the tumor, the tumor-draining lymph nodes, and in the spleen. We found that frequencies of SIY-reactive CD8^+ ^T cells increased in both the periphery and the tumor after these antibody combinations. To test if newly primed cells from the periphery were required for tumor regression, we used the S1P1 inhibitor FTY720 to block T cell egress from lymph nodes. FTY720 treatment given continuously beginning hours before these antibody combinations did not prevent tumor control, suggesting that the effects of combinations of agonist 4-1BB antibody with inhibitory antibodies to CTLA-4, PD-L1 or LAG-3 occur within the tumor microenvironment. Effective combinations restored IL-2 production by CD8^+ ^T cells within the tumor site. Our data suggest that reversing T cell dysfunction within the tumor microenvironment may be a common mechanism of multiple immunotherapy combinations, and that positive costimulatory signals can synergize with blockade of inhibitory receptors to reverse TIL dysfunction and support tumor regression.

